# Development of a Prediction Model for Community-Dwelling Older Adults at Risk of Long-Term Care with Dementia

**DOI:** 10.3390/geriatrics11020029

**Published:** 2026-03-05

**Authors:** Kana Kazawa, Ken Sugimoto, Yoko Aihara, Michiko Moriyama

**Affiliations:** 1 Graduate School of Biomedical and Health Sciences, Hiroshima University, Hiroshima 734-8553, Japan; morimich@hiroshima-u.ac.jp; 2 Department of General Geriatric Medicine, Kawasaki Medical School, Okayama 701-0192, Japan; ksugimoto@med.kawasaki-m.ac.jp; 3 Department of Nursing, Faculty of Health Sciences, Okayama University, Okayama 700-8558, Japan; ayohko99@okayama-u.ac.jp

**Keywords:** older adults, long-term care, dementia, predictive modeling

## Abstract

**Background:** Early detection of modifiable risk factors for long-term care certification with dementia is essential. This study aimed to develop a risk-scoring tool using data from the Kihon Checklist and Questionnaire for the Late-Stage Elderly over a 2-year period to predict long-term care certification with dementia under Japan’s Long-Term Care Insurance system. **Methods:** Participants included 2041 functionally independent, community-dwelling older adults in Kure City, Japan, as of March 2021. A retrospective cohort study was conducted. Associations between KCL and LSEQ domains and certification for long-term care with dementia were examined using logistic regression. To improve practical use, a score chart was developed to predict certification for long-term care with dementia. **Results:** Two years after completing the Kihon Checklist and Questionnaire, 143 participants (7.0%) were certified for long-term care with dementia. Factors independently associated with certification for long-term care with to dementia included age, homebound status, cognitive decline, and locomotor decline. The prediction model, developed using these variables, showed excellent discriminatory ability, with an area under the curve of 0.790 (95% confidence interval: 0.754–0.827). **Conclusions:** We developed an effective predictive model for future long-term care certification with dementia using routinely collected administrative data. This tool may help healthcare providers and health planners identify older adults at increased risk of long-term care certification with dementia.

## 1. Introduction

With the global aging of the population, the prevalence of dementia is increasing rapidly and presents a significant public health challenge. Cognitive decline can reduce independence [1], social interaction, and quality of life, and increase healthcare costs [2,3]. In Japan, healthcare costs for adults aged 65 and over are 3.8 times higher than those for adults aged 64 and under. Additionally, about 20% of older adults require some form of long-term care in their daily lives, and dementia is the leading cause of long-term care [4]. Addressing modifiable risk factors that contribute to long-term care needs due to dementia among older adults is a high priority for healthcare professionals and policymakers worldwide.

Longitudinal studies of dementia have identified potentially modifiable risk factors such as smoking, obesity, depression, physical inactivity, and excessive alcohol consumption [5,6]. Because these risk factors are interrelated, interventions targeting multiple factors are important. The FINGER study, the first of its kind globally, reported reduced progression of cognitive impairment after two years of observation [7]. Similar initiatives have been reported in Japan, including the J-MINT study [8,9].

To improve the effectiveness of multifactorial interventions tailored to individual risk factors, it is essential to identify individuals at high risk of future long-term care certification due to dementia and intervene early. In Japan, screening tools such as the Kihon Checklist (KCL) and the Late-Stage Elderly Questionnaire (LSEQ) have been introduced for comprehensive geriatric assessment and the screening of frailty. The KCL includes 25 questions that assess seven domains: difficulties in daily activities, decline in locomotor function, poor nutrition, decline in oral function, being homebound, decline in cognitive function, and depressive mood. The KCL is widely used in Japanese communities to identify individuals eligible for long-term care prevention projects [10,11,12]. The LSEQ was introduced in 2020 as part of health check-ups for older adults aged 75 years and over [13]. It includes 15 questions that assess 10 domains: health status, mental health, eating habits, oral function, weight change, exercise and falls, cognitive function, smoking, social participation, and social support. The three factors that do not overlap with the KCL (health status, smoking, and social support) are risk factors for adverse health outcomes and care dependency among older adults, not only those aged 75 years and over [14,15]. Consequently, a questionnaire combining the KCL and LSEQ may help to identify individuals at high risk of long-term care certification due to dementia more accurately than using either questionnaire alone.

Local governments have used these tools primarily to deliver community-based preventive services at the population level rather than to identify individuals at high risk. Comprehensive evaluation methods that combine KCL and LSEQ domains related to dementia-associated long-term care may identify high-risk individuals and support multifactorial, tailored interventions that potentially reduce care dependency and improve health outcomes among older adults.

This study aimed to develop a risk-scoring tool to predict the likelihood of long-term care certification with dementia among older adults living in the community.

## 2. Methods

### 2.1. Study Design

A retrospective cohort study was conducted.

### 2.2. Participants

The participants were older adults aged 65 years or older and were not certified as requiring long-term care, residing in Kure City, Hiroshima Prefecture, Japan, at the time of questionnaire implementation as of 2021. Kure City combined the KCL and LSEQ questionnaires and gave them to people aged 65 and over who visited the city hall for care prevention consultations or took part in local care prevention programs. Participation in the questionnaire was voluntary and unrelated to eligibility for consultation or preventive services. Individuals were informed that declining participation would not affect their access to care prevention consultations or municipal services.

These questionnaires were provided for secondary use in research purposes.

### 2.3. Measures and Data Collection

#### 2.3.1. Demographic and Baseline Characteristics

Age and sex were collected at the time of distribution of the KCL and LSEQ.

#### 2.3.2. Assessment Tools: Kihon Checklist and the Late-Stage Elderly Questionnaire

The seven domains of KCL have cut-off points that are useful for predicting LTC certification. We applied all seven domains [16,17]. For the LSEQ, we used a questionnaire covering three domains (health status, smoking, and social support), excluding items that overlapped with the KCL domains (Table S1).

#### 2.3.3. Definition of Long-Term Care Certification with Dementia

Japan’s Long-Term Care Insurance system is a universal, public insurance scheme for people aged 65 and over (as well as certain individuals aged 40–64 with age-related illnesses), funded through taxation and insurance premiums. In the Japanese long-term care insurance system, the required level of care is assessed using two indicators: the level of independence of people with disabilities, which reflects how physical disability affects daily life, and the assessment criteria for activities of daily living for older people with dementia, which reflects how dementia symptoms affect daily life. In this study, individuals certified as requiring long-term care with dementia were defined as those classified as Grade I or higher according to the assessment criteria for activities of daily living for older people with dementia. According to the long-term care insurance system, Grade I indicates that an individual exhibits dementia-related symptoms that interfere with daily life but can still live independently with some supervision or support. Higher grades indicate greater dependency.

Certification under the Long-Term Care Insurance system indicates eligibility for long-term care services and does not specify whether services are provided in the community or in institutional settings. Therefore, our outcome includes individuals receiving either home-based or facility-based long-term care services.

### 2.4. Statistical Analysis

Data are presented as means ± standard deviation or as frequencies and percentages. Baseline characteristics were compared between participants certified for long-term care with dementia and those without long-term care certification. The Student’s *t*-test or Mann–Whitney U test was used as appropriate after assessing normality.

Then, we developed a risk-scoring tool to predict certification for long-term care with dementia. First, because age was the only continuous variable that needed to be treated as categorical data in a score chart, we examined the relationship between age and certification for long-term care with dementia using a restricted cubic spline. Based on these results, we divided participants into three groups: 65–74 years, 75–84 years, and over 85 years. Second, to confirm the absence of multicollinearity, we examined Cramér’s V coefficients for correlations between the KCL and LSEQ domains and eliminated variables with coefficients > 0.3, indicating moderate correlation. Third, we performed a logistic regression analysis with certification for long-term care with dementia as the dependent variable, and each of the seven KCL domains and three late-elderly domains as independent variables. In a preliminary analysis to assess the contribution of KCL and LSEQ to predicting certification for long-term care with dementia, we conducted a logistic regression analysis, adjusted for age and sex, using backward elimination to identify the most parsimonious set of predictors for the final risk score chart. Next, to enhance clinical applicability, we presented the final model as a score chart, using rounded values of the shrunken regression coefficients.

We used the receiver operating characteristic (ROC) curve and calculated the area under the curve (AUC) to assess the model’s discriminative ability. Sensitivity and specificity were calculated using Youden’s index method. We performed statistical analyses using IBM SPSS Statistics (Version 26, Chicago, USA) and R (ver. 4.0.3), and considered *p* < 0.05 to be statistically significant.

### 2.5. Ethical Considerations

For insured persons, in accordance with the Kure City Personal Protection Regulations, this study was conducted as a joint research project between Hiroshima University and Kure City as part of the city’s healthcare policies. Kure City, as the medical and long-term care insurer, provided the data, and the analysis used an opt-out approach. Opt-out information was made publicly available on the Hiroshima University website. The ethics committee of Hiroshima University approved the study (No. E2022-0256).

## 3. Results

**Figure 1:**
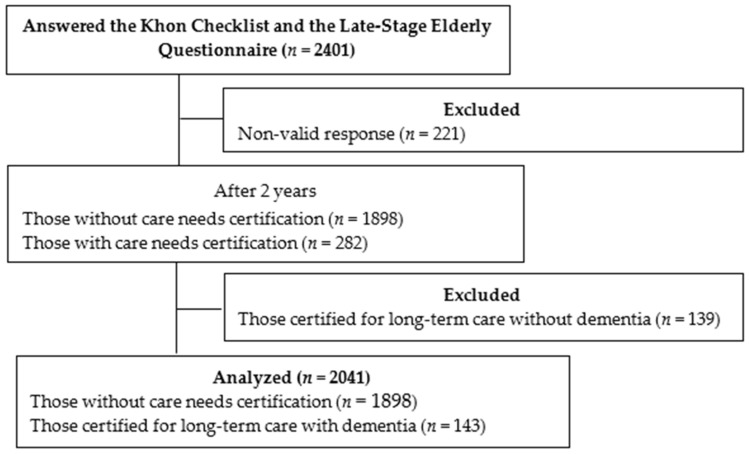
Flow diagram of the participants in this study.

Of the 2401 people who answered the questionnaire, 2180 responses were valid. After 2 years, 1898 did not receive long-term care certification. Among the 282 individuals certified as requiring care, 143 were identified as having dementia. For this analysis, we included only those without care needs certification (*n* = 1898) and those certified with dementia (*n* = 143), excluding those certified for long-term care without dementia (*n* = 139). Consequently, a sample of 2041 individuals was analyzed (Figure 1).

### 3.1. Baseline Characteristics of Participants

Table 1 presents a comparison of the attributes and responses for KCL and LSEQ. Individuals in the Certification group were significantly older than those in the Non-certification group (*p* < 0.001).

Regarding the KCL and LSEQ domains, the Certification for long-term care with dementia group had a higher proportion of individuals with locomotor function decline, homebound status, and cognitive decline than the Non-certification for long-term care group.

### 3.2. Predictors of Long-Term Care Certification with Dementia

Table 2 presents the results of a logistic regression analysis predicting certification for long-term care with dementia using backward stepwise selection. Age, decline in locomotor function, being homebound, and decline in cognitive function were statistically significant predictors of certification for long-term care with dementia. Age showed the highest odds ratio (85+ years: OR = 17.985, 95% CI = 9.257–34.942, *p* < 0.001; 75–84 years: OR = 4.301, 95% CI = 2.314–7.996, *p* < 0.001), followed by being homebound (OR = 2.643, 95% CI = 1.574–4.439, *p* < 0.001), decline of cognitive function (OR = 2.432, 95% CI = 1.669–3.545, *p* < 0.001), and decline of locomotor function (OR = 1.668, 95% CI = 1.123–2.479, *p* = 0.011). The calculated AUC was 0.791 (95% CI: 0.754–0.827).

### 3.3. Validation of the Risk Score Chart

**Figure 2:**
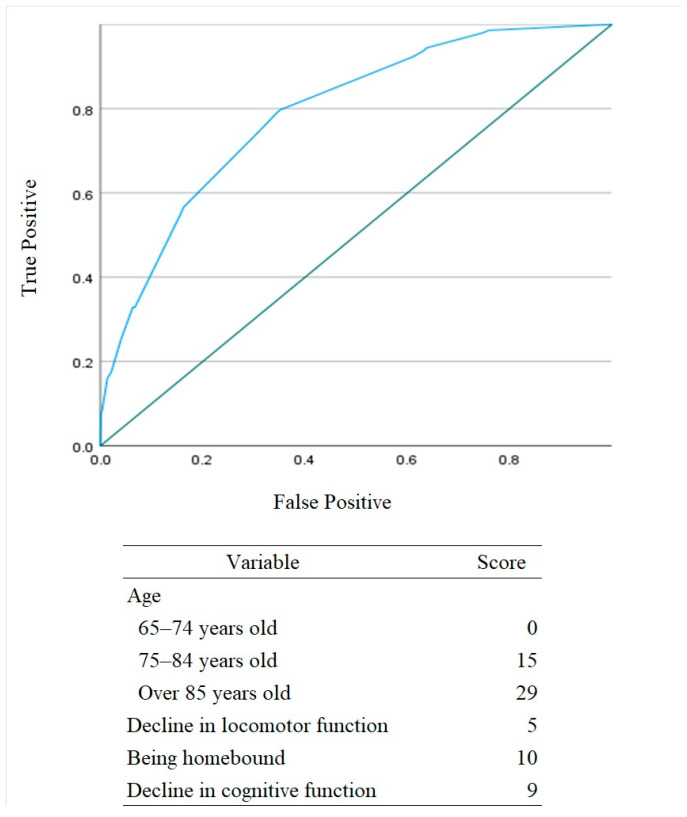
Receiver operating characteristic (ROC) curve for the risk evaluation model.

These four variables were used to create a score chart, with each variable’s score calculated based on its odds ratio (Figure 2). Using Youden’s index, the optimal cut-off point was 19. At this cut-off, sensitivity was 79.7%, and specificity was 64.9%, yielding the maximum sum of sensitivity and specificity. The AUC of the score chart was 0.790.

## 4. Discussion

### 4.1. Interpretation of Key Predictors

Only KCL factors were retained in the final model; LSEQ factors were not independently associated with certification. Age, homebound status, cognitive decline, and locomotor decline were independently associated with certification for long-term care with dementia. Age showed the highest odds ratio, indicating that advanced age was strongly associated with certification for long-term care with dementia [18].

Being homebound had the next highest odds ratio. Being homebound can reduce physical activity and cause muscle weakness [19], making it more difficult for individuals to leave their homes and participate in society [20]. Reduced communication with others lowers cognitive activity and interest in the environment. These changes can create a cycle that accelerates cognitive decline in older adults, making it more difficult for them to recognize the need to improve confinement and change their behavior [21], which may further reinforce homebound status. The background of being homebound includes physical factors such as pain, other symptoms, and impaired mobility; psychological factors such as impaired cognitive function and depression; and social factors such as economic deprivation and limited social participation [22]. A detailed, comprehensive assessment of these factors is necessary, followed by education, service introduction, and community development to address them. Previous research has shown that multiple domains of KCL are associated with future long-term care certification [11,23,24], and this study highlights the importance of early detection and intervention, especially for homebound status.

The third- and fourth-highest odds ratios were for cognitive and locomotor function decline, respectively. Subjective cognitive decline has been associated with subsequent dementia and an increased likelihood of long-term care needs [25,26]. However, studies have reported a delay between when individuals notice cognitive decline and when they receive a dementia diagnosis at a medical institution [27]. This delay may occur because individuals or their families interpret symptoms as normal aging [28,29] or because they fear, or are unable to accept, the possibility of cognitive decline or increased dependence in daily life [30]. Some causes of cognitive decline are treatable, and self-care and environmental adjustments can reduce the risk of decline and the need for care. It is necessary to provide accurate information about cognitive decline and dementia to community residents and to establish support systems that offer continuous assistance before and after diagnosis. A decline in physical function could lead to difficulties with everyday activities and social participation, reduced social interaction, and diminished motivation towards an active lifestyle. Being housebound, cognitive decline, and reduced locomotor function can reinforce each other, exacerbating frailty. A comprehensive intervention that addresses these multifaceted risks is essential [31].

### 4.2. Implications for Community-Based Risk Stratification and Intervention

From a community implementation perspective, this risk-prediction tool can serve as a practical link between epidemiological evidence and preventive care for older adults living in the community [5,32]. The tool may support population-level risk stratification, enabling local governments to allocate limited resources more efficiently by prioritizing high-risk subgroups. By enabling healthcare professionals and older adults to identify the risk of certification for long-term care with dementia and its associated risk factors, the tool may support targeted interventions within community-based long-term care prevention programs. For public health nurses and care managers, this tool may support structured outreach to homebound individuals, facilitate early referral to memory clinics, and guide individualized care planning within community-based integrated care systems. When combined with community resources such as exercise programs, cognitive stimulation activities, and social participation initiatives, the tool could promote coordinated, multi-sectoral preventive strategies. Visualizing changes in risk scores over time may also increase older adults’ motivation, health literacy, and sustained engagement in preventive actions, which remain key challenges in community-based prevention [33].

Furthermore, implementing this tool may help reduce disparities in access to preventive care by providing a standardized, scalable screening approach [34]. Future research should assess the efficacy of targeted interventions [35] and examine the feasibility, acceptability, and cost-effectiveness of using the tool in community settings [36]. This evidence is essential to inform policy decisions and support the sustainable integration of risk-prediction tools for long-term care certification with dementia into community-based care systems.

### 4.3. Study Limitations

This study has several limitations. First, because the data were collected at a municipal point of entry for care prevention consultations and programs, participants may have had higher health awareness than the general population, potentially introducing selection bias. Second, the model was developed using data from a single municipality and has not yet undergone external validation. Therefore, its generalizability to other regions or populations remains uncertain. Third, the risk score chart was developed using self-reported questionnaire data to support community-based care prevention activities without requiring special instruments or clinical assessment. Consequently, measurement error may be present, and the objectivity and reproducibility of the findings may be limited. Finally, the results of this study may underestimate risk, and some identified risk factors may not be modifiable.

## 5. Conclusions

We developed a risk-scoring tool to predict future long-term care certification with dementia among community-dwelling older adults using routinely collected administrative data. Early identification of high-risk individuals may facilitate targeted preventive interventions, population-level screening strategies, and timely risk communication with patients and families.

## Figures and Tables

**Table 1:** Comparison of factors between the Certification for long-term care with dementia group and the Non-certification for long-term care group.

Variables	Certification for Long-Term Care with Dementia Group (*n* = 143)		Non-Certification for Long-Term Care Group (*n* = 1898)		*p*-Value
Age					
65–74 years old	12	(8.4%)	756	(39.8%)	<0.001 ^a^
75–84 years old	78	(54.5%)	992	(52.3%)	
Over 85 years old	53	(37.1%)	150	(7.9%)	
Sex					
Men	119	(83.2%)	1536	(80.9%)	0.580 ^b^
Women	24	(16.8%)	362	(19.1%)	
Questionnaire domains					
Difficulties in daily activity ^§^	16	(11.2%)	51	(2.7%)	<0.001 ^a^
Decline in locomotor function ^§^	50	(35.0%)	299	(15.8%)	<0.001 ^a^
Poor nutrition ^§^	3	(2.1%)	17	(0.9%)	0.161 ^a^
Decline in oral function ^§^	34	(23.8%)	390	(20.5%)	0.392 ^a^
Being homebound ^§^	25	(17.5%)	98	(5.2%)	<0.001 ^a^
Decline in cognitive function ^§^	92	(64.3%)	732	(38.6%)	<0.001 ^a^
Depressive mood ^§^	64	(44.8%)	609	(32.1%)	0.002 ^a^
Poor health status ^§§^	17	(11.9%)	137	(7.2%)	0.048 ^a^
Smoking ^§§^	5	(3.5%)	49	(2.6%)	0.424 ^a^
Lack of social support ^§§^	6	(4.2%)	82	(4.3%)	1.000 ^a^

^a^ Mann-Whitney test, ^b^ Chi-square test. ^§^ Kihon Checklist domains, ^§§^ The Late-Stage Elderly Questionnaire items.

**Table 2:** Related factors for participants certified for long-term care with dementia.

Variables	Odds Ratio	(95% CI)	*p*-Value
Age			
65–74 years old	Reference		
75–84 years old	4.301	(2.314–7.996)	<0.001
Over 85 years old	17.985	(9.257–34.942)	<0.001
Decline in locomotor function	1.668	(1.123–2.479)	0.011
Being homebound	2.643	(1.574–4.439)	<0.001
Decline in cognitive function	2.432	(1.669–3.545)	<0.001

Cut-off points for the seven domains of KCL: difficulties in IADL (≥3 out of 5 questions); decline in locomotor function (≥3 of 5 questions); decline in cognitive function (≥2 of 3 questions). Multivariate logistic regression analysis using the backward stepwise selection method; Hosmer–Lemeshow test *p* = 0.559; AUC was 0.791 (95% CI: 0.754–0.827). CI confidence interval.

## Data Availability

The datasets presented in this article are not readily available because this study was conducted as the insurer’s project (Kure City) in accordance with the Personal Information Protection Ordinance.

## Supplementary Materials

The following supporting information can be downloaded at: https://www.mdpi.com/article/10.3390/geriatrics11020029/s1, Table S1: Questionnaires used in this study.

## Abbreviations

The following abbreviations are used in this manuscript:

KCL	Kihon Checklist
LSEQ	Late-Stage Elderly Questionnaire
