# Supplementary material for: Development of a Prediction Model for Community-Dwelling Older Adults at Risk of Long-Term Care with Dementia
Source: Geriatrics (Basel). 2026 Mar 5;11(2):29. doi: 10.3390/geriatrics11020029 (PMC13010682; doi:10.3390/geriatrics11020029)
Supplement: Supplementary file 1 [file geriatrics-11-00029-s001.zip › geriatrics-4144935-supplementary.pdf]

Table S1. Questionnaires used in this study

Item	Questions	Answer
1	Do you go out by bus or train by yourself?	0: Yes 1: No
2	Do you go shopping to buy daily necessities by yourself?	0: Yes 1: No
3	Do you manage your own deposits and savings at the bank?	0: Yes 1: No
4	Do you sometimes visit your friends?	0: Yes 1: No
5	Do your family or friends turn to you for advice?	0: Yes 1: No
6	Do you normally climb stairs without using handrail or wall for support?	0: Yes 1: No
7	Do you normally stand up from a chair without any aids?	0: Yes 1: No
8	Do you normally walk continuously for 15 minutes?	0: Yes 1: No
9	Have you experienced a fall in the past year?	1: Yes 0: No
10	Do you have a fear of falling while walking?	1: Yes 0: No
11	Have you lost 2kg or more in the past 6 months?	1: Yes 0: No
12	Height: cm, Weight: kg, BMI: kg/m2 If BMI is less than 18.5, this item is	1: Yes 0: No
13	Do you have any difficulties eating tough foods compared to 6 months ago?	1: Yes 0: No
14	Have you choked on your tea or soup recently?	1: Yes 0: No
15	Do you often experience having a dry mouth?	1: Yes 0: No
16	Do you go out at least once a week?	0: Yes 1: No
17	Do you go out less frequently compared to last year?	1: Yes 0: No
18	Do your family or your friends point out your memory loss? e.g."You ask the same question over and over again."	1: Yes 0: No
19	Do you make a call by looking up phone numbers?	0: Yes 1: No
20	Do you find yourself not knowing today's date?	1: Yes 0: No
21	In the last 2 weeks have you felt a lack of fulfillment in your daily life?	1: Yes 0: No
22	In the last 2 weeks have you felt a lack of joy when doing the things you used to enjoy?	1: Yes 0: No
23	In the last 2 weeks have you felt difficulty in doing what you could do easily before?	1: Yes 0: No
24	In the last 2 weeks have you felt helpless?	1: Yes 0: No
25	In the last 2 weeks have you felt tired without a reason?	1: Yes 0: No
26	How is your current health condition?	0: Good/ Fairly good/ Normal<br>1: Not so good/ Not so good
27	Do you smoke?	1: Smoke<br>0: Do not smoke/ Quit
28	Is there someone close to you that you can talk to when you are not feeling well?	0: Yes 1: No

Items No.1-25 were from the Kihon Check List and items No.26-28 were from the Late-Stage Elderly Questionnaire.

No. 1 to 25 (Kihon Check List) are judged to follow as:

1. Difficulties in daily activities: if 10 or more out of 20 items (No. 1-20) show a lower activity level.
2. Decline in locomotor function: if 3 or more out of 5 (No. 6-10) items show a lower level of physical strength.
3. Poor nutrition: if all the two items (No. 11, 12) show a lower nutritional status.
4. Decline in oral function: if two or more out of 3 items (No. 13-15) show a lower level of oral function
5. Being homebound: if the corresponding answer is yes to the question No.16
6. Decline in cognitive function: if one or more out of 3 items (No. 18-20) show a lower level of cognitive function
7. Depressive mode: if two or more out of 5 items (No. 21-25) are applied.
